# Conversion Surgery for Advanced Pancreatic Cancer

**DOI:** 10.3390/jcm8111945

**Published:** 2019-11-12

**Authors:** Thomas Hank, Oliver Strobel

**Affiliations:** Department of General, Visceral and Transplantation Surgery, Heidelberg University Hospital, Im Neuenheimer Feld 110, 69120 Heidelberg, Germany

**Keywords:** pancreatic cancer, conversion surgery, locally advanced pancreatic cancer, neoadjuvant therapy, FOLFIRINOX

## Abstract

While primarily unresectable locally advanced pancreatic cancer (LAPC) used to be an indication for palliative therapy, a strategy of neoadjuvant therapy (NAT) and conversion surgery is being increasingly used after more effective chemotherapy regimens have become available for pancreatic ductal adenocarcinoma. While high-level evidence from prospective studies is still sparse, several large retrospective studies have recently reported their experience with NAT and conversion surgery for LAPC. This review aims to provide a current overview about different NAT regimens, conversion rates, survival outcomes and determinants of post-resection outcomes, as well as surgical strategies in the context of conversion surgery after NAT. FOLFIRINOX is the predominant regimen used and associated with the highest reported conversion rates. Conversion rates considerably vary between less than 5% and more than half of the study population with heterogeneous long-term outcomes, owing to a lack of intention-to-treat analyses in most studies and a high heterogeneity in resectability criteria, treatment strategies, and reporting among studies. Since radiological criteria of local resectability are no longer applicable after NAT, patients without progressive disease should undergo surgical exploration. Surgery after NAT has to be aimed at local radicality around the peripancreatic vessels and should be performed in expert centers. Future studies in this rapidly evolving field need to be prospective, analyze intention-to-treat populations, report stringent and objective inclusion criteria and criteria for resection. Innovative regimens for NAT in combination with a radical surgical approach hold high promise for patients with LAPC in the future.

## 1. Introduction 

Pancreatic ductal adenocarcinoma (PDAC) is the fourth most common cause of cancer-related deaths and will become the second cause by 2030 [1,2,3]. This is due to a lack of specific symptoms and reliable screening tools resulting in tumor detection at advanced stages of disease, aggressive tumor biology and low chemosensitivity [4,5,6]. However, there has been progress, with an increase in survival rates after PDAC resection and adjuvant therapy with encouraging survival results especially in recent randomized trials testing multiagent adjuvant chemotherapy [6,7,8,9]. The main advances in pancreatic cancer surgery were achieved in patients with primarily resectable PDAC, which accounts for only 15–20% of all patients [6,10]. A larger subset of about 40% of patients present with locally advanced pancreatic cancer (LAPC) that was previously considered unresectable and an indication for palliative therapy [11]. LAPC is generally defined by local tumor growth with involvement (partial encasement of >180° or true infiltration) of the celiac axis or the superior mesenteric artery, according to the National Comprehensive Cancer Network (NCCN) [12], the Americas Hepato-Pancreato-Biliary Association (AHPBA)/Society of Surgical Oncology (SSO)/Society for Surgery of the Alimentary Tract (SSAT) [13] or the International Study Group of Pancreatic Surgery (ISGPS) guidelines [14]. Even if arterial resections, including the celiac axis and the superior mesenteric artery, are technically feasible, they may not be oncological reasonable because they are associated with high morbidity and mortality and prognosis is poor due to early local and systemic recurrence [15,16]. As long-term survival can be observed in individual cases, it has been suggested that arterial resection may be indicated in highly select cases [16].

In the last decade, a strategy of neoadjuvant therapy (NAT) followed by conversion surgery has been tested and is increasingly used in the treatment of primarily unresectable LAPC [17]. This strategy incorporates (i) the concept of a downstaging with NAT to a borderline-resectable or even resectable tumor that does not require an arterial resection, (ii) a biological selection of patients with response or at least without systemic progression during NAT pointing to less aggressive tumor biology, and (iii) an increased chance of achieving a true R0 resection associated with improved postresection survival [14,18]. Most of the early studies resulted from Gemcitabine-based combination therapies that were established for the palliative setting, and were then transferred to the “neoadjuvant” application for LAPC [19,20]. The introduction of the multi-agent chemotherapy regimen FOLFIRINOX in the palliative setting and its application for patients with LAPC marked an important advance in the field of conversion surgery after chemotherapy for LAPC [21,22,23]. Today, resection after NAT is increasingly used and has been reported in studies from around the world [24,25,26,27,28]. However, outcome data of these studies on NAT in PDAC has to be interpreted with caution because the available studies include heterogeneous populations with different disease stages ranging from clearly resectable, to borderline-resectable, locally unresectable, and even to primarily metastatic tumors [23,29,30]. Recently, several larger cohort studies on NAT and conversion surgery were published and provide new insights in this complex and fast-evolving field.

Here, we aim to provide an overview about current concepts of NAT and conversion surgery in advanced pancreatic cancer including different neoadjuvant regimens, patient selection for surgery, surgical techniques, postoperative management, and outcomes.

## 2. “Neoadjuvant” Therapy for Locally Advanced Pancreatic Cancer

While LAPC was previously considered an incurable disease stage and indication for palliative therapy along with metastatic PDAC, it is now increasingly recognized as a potentially curable disease. Several guidelines for LAPC have been published, although the evidence on its best treatment remains limited [12,31]. In contrast to upfront resections for resectable PDAC, several differences should be emphasized. In patients with LAPC, a biopsy of the tumor—which is not generally required for upfront resections—is mandatory before initiation of NAT [12,32]. In addition, pre-treatment laboratory workup should include CA19-9 levels to monitor disease response before and after completion of NAT [33,34,35]. If biliary obstruction is already present or likely to develop during the course of NAT, biliary stent placement is necessary before initiation of NAT [36]. After this initial workup is completed, patients with LAPC should promptly receive the most effective treatment regimen that can be administered based on their performance status as assessed by the Eastern Cooperative Oncology Group (ECOG)-score [37]. In patients with good performance status, defined as an ECOG-score from 0–1 together with an adequate nutritional status, the recommended first-line chemotherapy, equivalent to the guidelines for patients with metastatic PDAC, is either FOLFIRINOX or Gemcitabine in combination with Capecitabine or albumin-bound paclitaxel [21,38,39,40]. For patients with higher ECOG-scores, a Gemcitabine-based regimen is preferred as it is generally less toxic [12]. In Asian populations, patients may benefit from other neoadjuvant strategies, especially S1 an oral prodrug of 5-FU (tegafur) in combination with 5-chloro-2,4-dihydroxypyridine and potassium oxonate, which showed encouraging survival results in the metastatic and adjuvant setting [8,41]. Apart from these options, patients should be encouraged to participate in clinical trials on novel treatment regimens for LAPC [42].

In general, NAT is performed for almost 6 months, which equals six to eight cycles of chemotherapy and can be further extended in some patients, as demonstrated in the palliative situation [43]. In a multicenter study from Japan, it was shown that extended NAT was an independent predictor of overall survival in resected PDAC patients [44]. In a second study, prolonged NAT, as defined by ≥6 cycles of NAT, was confirmed as an independent predictor of increased overall survival [45]. However, the best duration of NAT remains to be determined and may also have to be adjusted to individual factors such as initial tumor burden, response evaluation and toxicity in prospective studies.

During NAT, the performance status, toxicity, and tumor response should be closely monitored. Cross-sectional imaging is usually performed every 2–3 months together with assessment of CA 19-9 levels as the current gold-standard biomarker for therapy monitoring [12]. Novel biomarkers, such as cell-free DNA tests to determine changes in KRAS mutation status during the course of NAT, have been developed as potential tools to determine biological tumor response in the future [46,47,48]. If patients present with disease progression, as indicated by local tumor growth or metastases in cross-sectional imaging and rising CA19–9 levels or with intolerable drug toxicity, a switch to a second-line chemotherapy regimen should be evaluated. For local tumor progression, chemoradiation with Capecitabine or 5-FU plus concurrent radiotherapy may be considered as additional therapy [49,50].

Local disease response in cross-sectional imaging is not necessary to qualify for conversion surgery, as radiological restaging can be misleading after NAT in PDAC [19,51,52]. Therefore, all LAPC patients without signs of disease progression, such as newly diagnosed metastasis, rising CA19–9 levels, or with inadequate performance status, should be evaluated for conversion surgery by a multidisciplinary board.

## 3. Conversion Surgery after Neoadjuvant Therapy for Advanced Pancreatic Cancer

Conversion surgery after NAT has been performed and reported in small observational studies for many years. In 2010, a systematic review reported response and resectability rates in NAT patients [10]. The overall population included 46% of patients who had been classified as having initially unresectable tumors. The majority of these patients received 5-FU- or Gemcitabine-based regimens, since FOLFIRINOX had not yet been introduced. Complete response was reported in 4.8% and partial response in 30.2% of patients. In borderline-resectable and LAPC, a resectability rate of 33% was reported. Interestingly, the median survival in this group was comparable to that of upfront resectable patients with 20 vs. 23 months, respectively. Shortly afterwards, a study reported comparable median survival of 25 months in 36 patients with unresectable PDAC receiving NAT in a matched group of patients with primarily resectable tumors [53]. The largest study on NAT in the pre-FOLFIRINOX era was published by the Heidelberg group in 2012 and included 257 LAPC patients undergoing exploration after NAT with a resectability rate of 46% [19]. Of 120 patients with resection, 47 (39%) received extended resections and 45 (37%) had vascular resections. In 36 (30%) patients with a R0 resection, median postoperative survival was 23 months compared to only 9 months after surgical exploration.

The introduction of the FORFIRINOX regimen considerably advanced the treatment of LAPC and of conversion surgery. A patient-level meta-analysis investigating the outcomes in LAPC patients receiving first-line therapy with FOLFIRINOX included 315 patients from 11 observational studies [22]. The median overall survival was 24 (range 10–32) months and the median progression-free survival was 15 (range 3–24) months. The number of NAT cycles varied from 3 to 11 across the studies with frequent dose modifications and dose reductions. Almost 65% of the patients received additional radiotherapy or radio-chemotherapy after completion of FOLFIRINOX. The percentage of conversion surgery ranged from 0–43% with a pooled percentage of 26% and an R0 rate between 50–100%, overall representing a highly heterogeneous approach to the management of LAPC across studies. Of note, these resection rates are much lower compared to a recent patient-level meta-analysis including 313 borderline-resectable patients [54]. Janssen et al. analyzed 24 studies comprising 313 patients following FOLFIRINOX with a resection rate of 67.8% (95% CI, 60.1–74.6) and an R0-rate of 83.9% (95% CI, 76.8–89.1). Median overall and progression-free survival after treatment initiation from patient-level data for 283 individuals was 22.2 and 18 months, respectively. 

Several larger observational studies on LAPC patients were published more recently (Table 1). The Heidelberg group reported on 575 patients undergoing exploration after NAT for LAPC (76%) or metastatic PDAC (24%) [23]. Most patients received neoadjuvant Gemcitabine-based regimens (56%), followed by FOLFIRINOX treatment (21.7%). Patients who underwent surgical exploration had successful tumor resection in 51%, requiring extended resections in one-third of patients. The highest resection rate was achieved after NAT with FOLFIRINOX, resulting in a resection rate of more than 60% of patients undergoing surgery. The median survival in the entire cohort was 15.3 months after surgical resection compared to 8.5 months when receiving palliative treatment. While interpreting this survival outcome, it must be kept in mind that this study included no borderline-resectable tumors, but only truly local, advanced, unresectable tumors and more importantly, 24% metastatic tumors. A study from Johns Hopkins University reported their experience in 415 patients that fulfilled the criteria for LAPC [55]. Of patients who completed ≥4 months of NAT, 116 individuals (28%) were scheduled for surgical exploration. Of those, 84 patients (78%) underwent successful resection, representing 20% of the entire LAPC cohort receiving NAT. The majority of patients with successful conversion surgery received FOLFIRINOX treatment (60%) followed by Gemcitabine regimens (19%) and a combination of both regimens. In contrast, non-resected patients were more likely to receive Gemcitabine-based therapy, partly explained due to a lower ECOG-performance status. In addition, non-resected patients had higher CA19–9 levels and larger tumors at cross-sectional imaging. Median overall survival was significantly higher in resected patients with 35 months compared to 16 months in non-resected patients. These favorable survival data are similar to the findings from Massachusetts General Hospital. In their study on NAT with FOLFIRINOX, 51% of patients had LAPC and 49% had borderline-resectable tumors. Median overall survival for the 110 patients that underwent resection was 37.7 months after initial diagnosis and 31.5 months after surgery compared to 18.6 months in non-resected individuals [30]. More recently, a Phase II clinical trial was reported from the same institution, including 49 LAPC patients that received FOLFIRINOX in combination with Losartan as NAT [56]. Some 42 (86%) patients underwent surgical exploration with a final conversion rate of 69% and a R0-rate of 61%. Median survival was 31 months in the entire cohort and 36 months in patients undergoing surgical resection. In 2019, the Verona group reported a prospective study on intended NAT and conversion surgery with an intention-to-treat (ITT) analysis [57]. The authors applied very liberal inclusion criteria for a final cohort of 680 patients of which 60.7% were diagnosed with LAPC and 39.3% with borderline-resectable tumors. The treatment completion rate in the entire cohort was 71.6%. Most patients received FOLFIRINOX treatment (45.6%) followed by Gemcitabine plus nab-paclitaxel (21.6%). In the ITT analysis, only every fourth patient underwent surgical exploration with a conversion rate of 15%. Subgroup analysis revealed resection rates of 24% in borderline-resectable patients but only 9% in LAPC. Resection was more likely in younger patients (≤75 years) with borderline-resectable disease and receiving FOLFIRINOX treatment. Median disease-specific survival for the entire cohort was 12.8 months. In patients who underwent surgical resection, the corresponding disease-specific median survival times were 35.4 and 41.8 months for borderline or LAPC subgroups, respectively.

The overview provided in Table 1 demonstrates overall promising results but point to a considerable heterogeneity among available studies as far as inclusion/resectability criteria, neoadjuvant regimens used, criteria for surgical exploration after NAT, extent of surgery, and as a consequence, resectability rates and survival outcomes, are concerned. While some of the available studies do not report on an ITT population entering a NAT strategy, the studies that provide such data report resectability rates that are overall much lower compared with the non-ITT reports. To advance the field, future studies on this topic should be designed as prospective studies with an ITT analysis, with strictly predefined and objective criteria for (un)resectability, response evaluation during NAT, selection criteria for conversion surgery, and indications for extended resections. More Phase-2 studies on innovative combination therapies are warranted to further advance the field. One major challenge is the fact that resectability criteria for PDAC may be nicely defined by different guidelines [12,13,14], but their standardized application is considerably hindered by subjective decisions based on personal experience and preferences of surgeons, radiologists and oncologists participating in multidisciplinary tumor boards. In a recent study, the agreement between 7 multidisciplinary tumor boards in both resectability evaluation and treatment allocation in potentially resectable pancreatic cancer was below 50% in patients with localized PDAC [66]. Therefore, more objective tools for pretherapeutic prediction of resectability and prognosis that can be assessed in a standardized fashion around the world are urgently needed [6,67].

## 4. Conversion Surgery for Metastatic PDAC 

The majority of the literature on conversion surgery examines resections after NAT for localized PDAC. However, there is some sparse data on conversion surgery after chemotherapy for metastatic PDAC, as shown in Table 2. The Heidelberg study included 135 (23.5%) patients undergoing surgical exploration after NAT for initially metastatic disease and 51 patients (17.5% of patients undergoing resections) had limited metastatic disease at the time of resection in the liver (69%), peritoneum (17%), or adrenal glands (14%) [23]. While metastatic disease was an independent predictor of shorter survival after resection and exploration, survival outcome was not separately reported for this subgroup. Another report from two US institutions demonstrated a favorable median overall survival in 23 patients with metastatic PDAC of 18 months after conversion surgery and 34 months after initial diagnosis [68]. Most patients received FOLFIRINOX treatment with a median duration of 9 cycles and surgical approaches included extended resection with metastasectomy. In a study on patients with synchronous liver metastases, 24 (4.5%) of 535 patients underwent secondary resection [69]. Selection criteria for surgical exploration were the disappearance of liver metastasis on pre-operative imaging and favorable CA19–9 response. Median time from diagnosis to surgery was 9 months and FOLFIRINOX was the preferred first-line therapy. Pancreatic resection was finally performed in 24 patients without evidence of residual metastatic lesions assessed by intraoperative ultrasound of the liver and was associated with a median overall survival of 56 months after initial diagnosis. Overall, these data point to a selection of “super-responders” to NAT. A Phase II trial even reported on conversion surgery in patients presenting with peritoneal carcinomatosis [70]. In this Phase II trial, 33 patients with macroscopic peritoneal metastasis or positive cytology received oral S1 in combination with intraperitoneal/i.v. Paclitaxel for a median duration of 8.8 months. Some eight patients (24%) underwent conversion surgery with a median survival of 27.8 months vs. 14.2 months in patients without resection. Criteria for conversion surgery were negative cytology, decreasing CA19–9 levels and disappearance of macroscopic tumor dissemination assessed by staging laparoscopy.

Taken together, these studies mostly report favorable results of conversion surgery in highly selected “super-responders” to long-term chemotherapy. As in all studies on NAT, it remains unclear to what extent favorable survival outcomes are based on individual patient selection and/or surgical resection. Based on those reports with larger stage IV cohorts, conversion rates in an ITT population remain below 5% for this particular subgroup. With the increasing use of more effective chemotherapy regimens, more data on conversion surgery in metastatic patients will become available and will need to be carefully examined.

## 5. Techniques for Conversion Surgery after Neoadjuvant therapy

**Figure 1:**
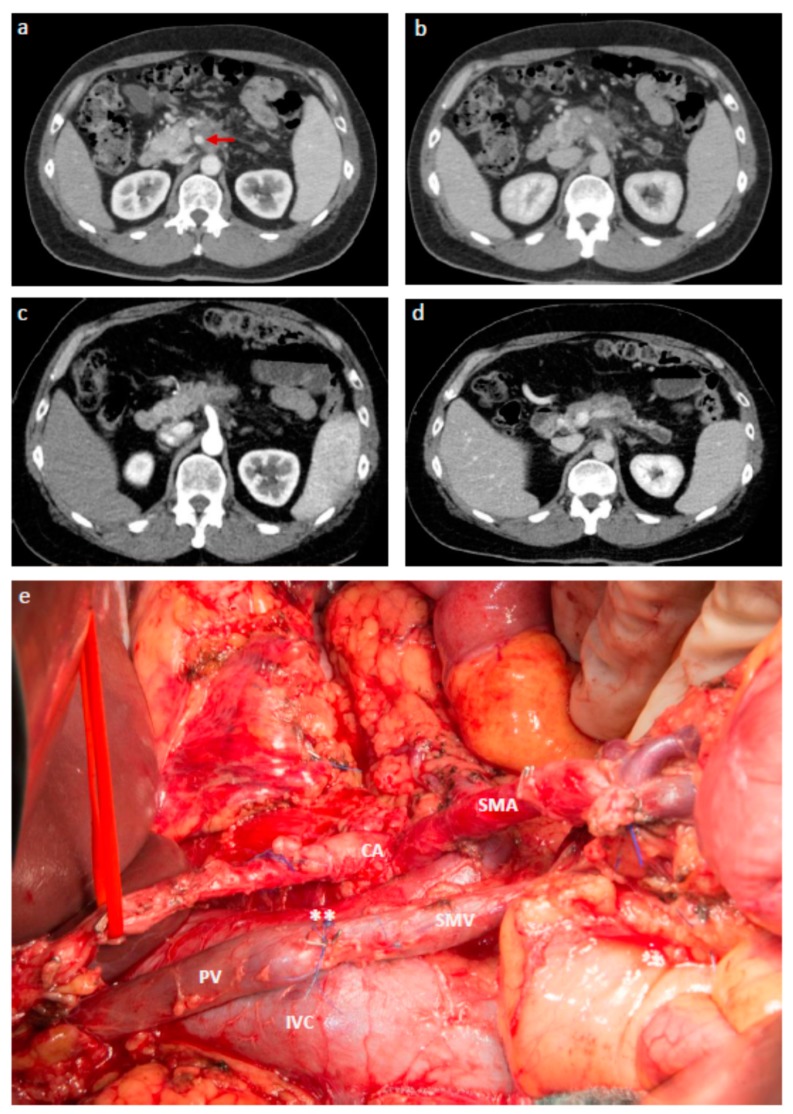
LAPC with partial response after NAT receiving extended tumor resection with perivascular dissection: a–d: Transverse multidetector contrast enhanced CT images taken before initiation of NAT ((**a**) arterial phase; (**b**), venous phase) and after completion of NAT with three cycles of FOLFIRINOX and 11 cycles of FOLFIRI (**c**, arterial phase; **d**, venous phase). Unresectable PDAC of the pancreatic body with encasement of the SMA (red arrow in a) and cavernous transformation of the portal vein (**b**). Partial response on CT (**c** and **d**). (**e**): Operative site after extended tumor resection with artery first, total pancreatectomy, splenectomy, adrenalectomy, subtotal gastrectomy, subtotal colectomy, TRIANGLE-operation with PV/SMV resection (**) and perivascular level-III dissection around the SMA and celiac axis (CA). Final pathology showed ypT2, ypN1 (1/49), L0, V0, R0. The patient had a peritoneal recurrence at 24 months after resection.

**Figure 2:**
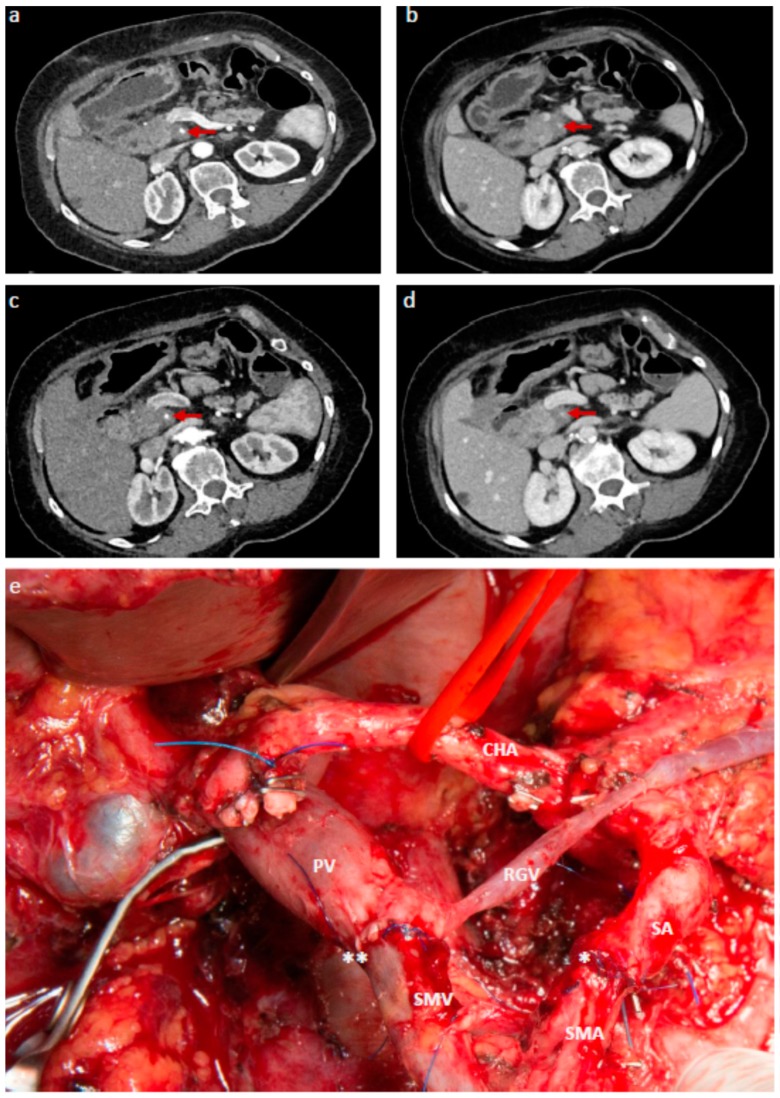
LAPC with stable disease after NAT receiving extended tumor resection with SMA resection: a-d: Transverse multidetector contrast enhanced CT images taken before initiation of NAT (**a**, arterial phase; **b**, venous phase) and after completion of six cycles of Gem-based NAT (**c**, arterial phase; **d**, venous phase). Unresectable PDAC in the pancreatic body with 360° encasement of the superior mesenteric artery (SMA, red arrow) and occlusion of the superior mesenteric vein (SMV). Stable disease on CT after 5 months (**c** and **d**). (**e**): Operative site after extended tumor resection with the artery-first approach, total pancreatectomy, splenectomy, subtotal gastrectomy, right hemicolectomy, resection of the portal vein (PV) and SMV with direct end-to-end anastomosis (**). SMA resection was performed with transposition of the splenic artery (SA), indicated with a single asterisk. Final pathology revealed ypT4, ypN2 (4/54), V1, L1, R1 with no evidence of disease 19 months after surgery.

In recent decades, surgical procedures for resectable PDAC were continuously refined and, in combination with more effective adjuvant chemotherapy regimens, markedly improved survival outcomes [6]. Surgical techniques aimed at local radicality are important for upfront resections, but probably even more in the context of conversion surgery for LAPC [71]. In the early 1990s, the concept of “isolated pancreatectomy” was introduced by Nakao et al. with the aim to radically resect pancreatic head cancers in an upfront surgery setting [72]. This concept included an artery-first approach, known as the mesenteric approach [73], with dissection at the superior mesenteric artery even before mobilization of the pancreatic head aiming at better vascular control, lower blood loss, higher R0 rates and improved overall survival [73,74]. Besides, several different artery-first approaches have been described that may have specific advantages dependent on surgical anatomy with exact location of the tumor and its relation to the superior mesenteric artery or the celiac axis [74,75]. An artery-first approach allows for assessment of local resectability early—during surgical exploration—before a point of no return is passed, thus, decreasing the risk of R2 resections and probably increasing the chance for R0 resection [74,75]. These potential advantages of the artery-first approach were supported by a meta-analysis including 16 retrospective studies with 771 artery-first maneuvers compared to 701 standard resections in the upfront setting [76]. To generate high-level evidence on this important topic, a multicenter randomized controlled trial of the mesenteric versus a standard approach during pancreatoduodenectomy for resectable and borderline-resectable PDAC is currently being conducted in Japan [77]. Given the putative advantages of an artery-first approach during upfront resection, these techniques are likely even more important in the context of conversion surgery after NAT for LAPC (Figure 1). This is based on two important aspects in this setting: First, these patients, by definition, initially present with tumors involving major arteries and even after NAT, local resectability remains defined by arterial involvement which, therefore, should be assessed early during exploration [23]. Second, this becomes even more relevant, as radiological resectability criteria including assessment of arterial involvement are no longer applicable after NAT [19,23,54]. Instead, local resectability can only be assessed by surgical exploration, with frozen section biopsies at the arteries [78]. If the frozen biopsy reveals a persistent true invasion of a major artery, surgical resection can be abandoned or the decision for an arterial resection has to be made (Figure 2). While upfront arterial resections should probably be avoided considering the balance between perioperative risk and potential long-term benefits [16], the role of arterial resections after NAT remains to be defined. If frozen biopsy reveals that only fibrotic tissue is present around the artery, one can usually find a layer directly between the vascular wall and the (previously infiltrated) perivascular plexus and perform a radical and macroscopically complete tumor resection. The extent of such a resection is in keeping with a radical perivascular level-3 dissection (according to Inoue) along the arteries and a total mesopancreatic excision [79] (Figure 1). In resectable PDAC, it is recommended that this radical dissection is performed at 180° at the tumor-oriented side of the vessels. During conversion surgery after NAT for a LAPC that by definition used to involve >180° up to total encasement of an artery, a complete circumferential clearance of the vessels is frequently necessary in order to radically remove the entire tumor tissue together with post-treatment fibrotic tissue. This radical resection technique results in an operative site in which the celiac axis/hepatic artery, the superior mesenteric artery and the venous porto-mesenteric axis form a triangle. The initial perioperative outcomes of this TRIANGLE operation were reported in a Phase 1 study from Heidelberg focusing on safety and feasibility of this technique. In 15 patients, major morbidity occurred in 46.6%, with a 30-day mortality of 0% and the R0 resection rate (based on the strict definition of a 1 mm free margin) was 40% [78]. However, larger studies on this radical technique and data on oncologic outcomes are necessary. While this radical resection technique frequently allows for complete macroscopic resections of LAPC after NAT without the risk of arterial resections, venous resections are usually still necessarily [6].

Body and tail cancers infiltrating the celiac trunk represent a special subgroup of LAPC, because in these tumors, a distal pancreatectomy with celiac axis resection (DP-CAR) without arterial reconstruction can be considered [80,81]. While DP-CAR resections are applied in the upfront setting, reported mortality is high, with 16%, and median survival times are sobering with only 18 months [80]. In spite of the technical feasibility of upfront DP-CAR, a neoadjuvant strategy may appear more promising in these tumors. An observational study on resection after NAT in a cohort of 31 patients with involvement of the celiac axis reported an R0 resection rate of 73% [82]. Severe complications were reported in 42%, with an in-hospital mortality of only one patient. Patients with NAT had a median survival after treatment initiation of 38.6 months with Gemcitabine + S1 and 19 months after Gemcitabine plus Paclitaxel [82]. In addition, a neoadjuvant strategy in these tumors can be combined with common hepatic/celiac axis embolization in order to enhance the formation of arterial collaterals and to thereby increase the safety of later celiac axis resection [83].

Taken together, the surgical strategy during conversion surgery after NAT for LAPC should include an artery-first approach for assessment of resectability early during exploration, in order to avoid R2 resections, and to achieve local radicality (R0 is the aim, but R1 resections may be acceptable). The plane of dissection should be directly at the vascular walls. Surgeons performing such conversion surgery should be well prepared to perform vascular resections. While venous resections are still necessary to achieve local radicality, arterial resections might be avoided. In patients with tumors still infiltrating an artery after NAT, the role of arterial resections needs to be further examined. These operations are technically demanding and should be performed in experienced centers.

## 6. Perioperative Outcome and Pathological Challenges

Several studies investigated morbidity of conversion surgery after NAT for LAPC with overall indecisive results—likely due to heterogeneous treatment algorithms [84]. Larger studies have recently described (major-) morbidity rates between 23% and 59% in borderline and LAPC patients [61,85,86,87]. In a comparative study, overall complications were significantly lower after resection following NAT with FOLFIRINOX compared to a control group of upfront resections (36% vs. 63%) [51]. Marchegiani et al. described similar morbidity rates after pancreatic head resections performed upfront and following NAT of 57.8% and 58.6%, respectively, but clinically relevant pancreatic fistula occurred significantly less often after NAT (9.1% vs. 15.1%) [88]. Moreover, by applying a clinical burden score, complications appear to be associated with an increased clinical burden if they occur after NAT compared to upfront resections, resulting in prolonged hospitalizations [88]. Another study demonstrated a 3.6-fold decrease of clinically relevant pancreatic fistula in 364 patients undergoing pancreatic resection following NAT compared to a contemporaneous control group of 407 patients receiving upfront resections [89]. The authors observed a change in classical determinants of pancreatic fistula in the NAT setting; only soft pancreatic tissue remained associated with the occurrence of pancreatic fistula. While pancreatic fistula did not impact median overall survival after upfront resections (26 with vs. 25 months without) it was associated with a significantly reduced overall survival in NAT patients (17 vs. 34 months) [89].

Another important aspect is the prognostification of patients after NAT and conversion surgery for LAPC. Currently, there are no valid clinical tools available to identify patients at high risk for early recurrence and early death after NAT. The role of additional adjuvant therapy after NAT and conversion surgery is unknown and there is no evidence for a standard of postoperative cancer-directed treatment. However, a relevant fraction of NAT patients has early recurrence or die within 12 months after conversion surgery [30,55]. Identification of these patients is clinically relevant as it appears likely that they would benefit from additional adjuvant therapy. Currently, the use of adjuvant therapy depends on a variety of factors, including local preferences of care-providers, individual performance status of patients and their ability to complete a (total-) NAT before surgery, as well as postoperative morbidity. Studies on the topic of prognostification after NAT and conversion surgery for LAPC and on the role of adjuvant therapy are urgently needed.

An important question in this context is if and how NAT impacts the prognostic relevance of clinical and pathological factors that are well-established predictors of survival after upfront resections. These classic predictors include tumor-related factors, such as pT-stage, tumor size, pN-stage, resection margin status, as well as CA19–9 levels [90,91,92,93]. Response to NAT considerably impact these parameters, resulting in smaller tumors, less lymph node involvement, and higher R0 rates when compared to upfront resections [89]. It is well documented that NAT causes extensive pathological changes in the pancreatic gland, resulting in a higher extent of fibrosis and pancreatic atrophy [94]. This can result in challenges for pathologists in defining regression grades for PDAC after NAT [95] or result in an overestimation of R0-rates since sparse tumor cells may skip the resection margin [96]. In one recent study on NAT patients, only tumor size remained an independent predictor of overall survival following surgery [30]. In another study, CA19–9 levels, lymph node involvement, metastatic disease, and vascular involvement were predictive of long-term survival after conversion surgery following NAT [85]. However, the strict R-status requiring a 1 mm free margin—an important independent predictor of survival in the upfront setting [92,93]—was associated with overall survival by univariable analysis, but was not confirmed as an independent predictor of survival by multivariable analysis, probably due to a heterogenous study population, including patients with metastatic disease [85].

In other studies, a good biological response, indicated by the course of CA19–9 levels, was found to be independently predictive for overall survival, along with a major pathological response defined by rare or absent viable tumor cells [34,45,65]. Perri et al. accessed predictive factors for major pathological response in NAT patients [97]. The authors identified an optimal CA19–9 response and partial radiological response according to the RECIST criteria as predictors for major pathological response. In line with these findings, data on patients with complete pathological response following NAT, which occurs in a small subset of 3–10% of patients receiving NAT [65,87,97], demonstrate exceptionally good survival outcomes with median overall survival of more than 60 months after conversion surgery compared to 26 months without a complete pathological response [87].

## 7. Conclusions

The strategy of NAT and conversion surgery for patients with LAPC is rapidly evolving, especially since more effective combination chemotherapies have become available. This is reflected by an increasing number of studies investigating this topic with heterogenous short- and long-term outcomes. In highly heterogeneous studies, the reported surgical conversion rates for LAPC vary between 9–87%. These numbers should be interpreted with caution since ITT analyses are lacking in most studies on this topic. Even in the few studies providing an ITT analysis, resectability rates vary extremely with variations in local practices of indication for resection and use of extended resections. Furthermore, survival data are difficult to read since the anatomical inclusion criteria for borderline-resectable or LAPC subgroups differ enormously between centers and studies. Future studies should try to incorporate objective biological criteria for resectability and response evaluation, instead of relying only on radiological criteria, which are often applied in a subjective and non-standardized fashion [65]. Of note, radiological resectability criteria are not applicable after NAT and experienced high-volume centers, therefore, surgical exploration in all patients without signs of tumor progression during NAT is recommended [6,54]. Overall, R0-rates in two-thirds of patients and median survival times of more than 35 months after treatment initiation and conversion surgery can be expected. Conversion surgery after NAT for LAPC is demanding and requires advanced surgical strategies and techniques, including artery-first approaches, radical perivascular dissections, and extended vascular and multivisceral resections to achieve satisfying survival outcomes.

## Figures and Tables

**Table 1:** Overview of neoadjuvant chemotherapy regimens and resection rates for advanced pancreatic cancer.

Author	Year	Study Design	Patients undergoing NAT	Patients undergoing exploration	Study period	BR	LAPC	M1	NAT Protocol	Cycles NAT (*n*)	Duration NAT ^§^	Progressive Disease	Conversion/Resection Rate	Extended Resection	R0-rate	Median OS ^*^
Chang [58]	2011	Uni-centric, prospective, Phase II	50 ^#^	NA	2004–2008	4%	96%	0%	Gem-5-FU, GEM-RT	6	-	28%	8% ^#^	NA	-	14.5 months ^E^
Mukherjee [59]	2013	Multi-centric, prospective, Phase II	74 ^#^	NA	2009–2011	0%	100%	0%	GEM-RT, CAP-RT	-	12	-	6.7% ^#^	NA	100%	14.6 months ^E^
Youl [60]	2014	Uni-centric, retrospective	90 ^#^	NA	2001–2009	18%	82%	0%	GEM+ GEM-RT	6	-	23%	15.5% ^#^ 1.3% (LAPC)	NA	14.2%	12.7 months ^E^ 18.2 months ^S^
Sadot [61]	2015	Uni-centric, retrospective	101 ^#^	35 (34.7)	2010–2013	0%	100%	0%	FFX +/-Chemo-RT	6	13	5%	31% ^#^	NA	55%	25 months ^E^ not reached ^S^
Marthey [62]	2015	Multi-centric, prospective	77 ^#^	NA	2010–2012	0%	100%	0%	FFX +/-Chemo-RT	5	-	16%	36% ^#^	NA	89%	22 months ^E^ 24.9 months ^S^
Ferrone [51]	2015	Uni-centric, retrospective	NA	47	2011–2014	37.5%	62.5%	0%	FFX +/-Chemo-RT	8	-	-	85.1%	12.5% (venous resection)	92%	34 months ^E^
Hackert [23]	2016	Uni-centric, retrospective	NA	575	2001–2015	0%	76.5%	23.5%	FFX, GEM +/-RT, 5-FU	-	20 (GEM) 28 (FFX)	-	50.8% ^†^ 61% (FFX) 48% (GEM)	33% (venous, arterial, MVR)	23.6%	22.5 months (FFX) ^S^ 21.2 months (GEM) ^S^ 13.5 months (non-resected)
Khushman [63]	2016	Bi-centric, retrospective	51 ^#^	NA	2008–2013	22%	78%	0%	FFX +/-Chemo-RT	8	-	4%	22% ^#^	NA	95%	35.4 months ^E^
Hammel [50]	2016	Multi-centric, prospective, Phase III	449 ^#^	NA	2008–2011	0%	100%	0%	Gem +/-Erlotinib + GEM+/-RT	-	16 + 8	-	4% ^#^	NA	61%	12.8 months ^E^ 30.9 months ^S^
Michelakos [30]	2017	Uni-centric, retrospective	NA	141	2011–2016	49%	51%	0%	FFX +/-Chemo-RT	8	-	-	78%	NA	81%	34.2 months ^E^ 37.7 months ^S^
Gemenetzis [55]	2018	Uni-centric, retrospective	461 ^#^	116 (28%)	2013–2017	0%	100%	0%	FFX, FFX-GEM, GEM +/-RT	-	20	6%	20% ^#^ 63% (FFX) 17% (FFX-GEM) 20% (GEM)	27% (DP-CAR)	89%	35.3 months ^S^ 16.2 months (non-resected)
Reni [64]	2018	Multi-centric, prospective, Phase II	54 ^#^	NA	2014–2016	38% (PAXG) 54% (AG)	62% (PAXG) 46% (AG)	0%	AG, PAXG	5	24	0% (PAXG) 21% (AG)	0% (LAPC) ^#^ 31% (BR, PAXG) 32% (BR, AG)	23.5% (venous resection)	53%	19.1 months (PAXG) ^E^ 20.7 months (AG) ^E^
Macedo [65]	2019	Multi-centric, retrospective	NA	NA	2010–2016	46.4%	25.5%	0%	FFX, Gem+ Nab-Placitaxel	5 (FFX)3 (Gem)	-	-	Reports only resected patients (*n* = 274)	34.7% (venous resection)	82.5%	30.1 months BR ^S^ 33.1 months LAPC ^S^
Murphy [56]	2019	Uni-centric, prospective, Phase II	49 ^#^	42 (86%)	2013–2018	0%	100%	0%	FFX+ Losartan+/-Chemo-RT	8	-	10%	69% ^#^	14.7%(venous) arterial resection)	88%	31.4 months ^E^ 33 months ^S^
Maggino [57]	2019	Uni-centric, prospective	680 ^#^	147 (23.9%)	2013–2015	39.3%	60.7%	0%	FFX, Gem+ Nab-Placitaxel, GEMOX, GEM +/-RT	6	-	38%	15.1% ^#^ 9% (LAPC) 24.1% (BR)	27.8% (venous, arterial resection)	57.8%	12.8 months ^E^ 35.4 months BR ^S^ 41.8 months LAPC ^S^

NAT, neoadjuvant therapy; ^#^, intention-to-treat population (ITT); ^§^, weeks; *, median overall survival after diagnosis; BR, borderline-resectable pancreatic cancer; LAPC, locally advanced pancreatic cancer; M, metastatic pancreatic cancer; FFX, FOLFIRINOX; GEM, Gemcitabine-based regimen; RT, radiotherapy; CAP, Capecitabine; GEMOX, Gemcitabine and oxaliplatin; AG, nab-paclitaxel plus gemcitabine; PAXG, cisplatin, nab-paclitaxel, capecitabine and gemcitabine; MVR, multi-visceral resection; DP-CAR, distal pancreatectomy with celiac axis resection; E, Entire cohort; S, Surgical resected cohort; ^†^, all NAT regimens; ¶, Disease-specific survival.

**Table 2:** Overview of chemotherapy regimens and conversion resection rates for metastatic pancreatic cancer.

Author	Year	Study Design	Patients Undergoing NAT	Patients Undergoing exploration	Study Period	HEP	PUL	PER	NAT Protocol	Cycles NAT (*n*)	Duration NAT ^§^	Progressive Disease	Conversion/Resection Rate	Extended Resection	R0-rate	Median OS
Wright [68]	2016	Bi-centric, retrospective	1147 ^#^	NA	2008–2013	69%	26%	8%	FFX, Gem +/- RT	9	-	-	2% ^#^	47.8% (Metastatic resection *, RFA ^$^) 13% (venous resection)	91.3%	34.1 months ^S^
Satoi [70]	2016	Multi-centric, Prospective, Phase II	33 ^#^	NA	2012–2015	0%	0%	100%	Paclitaxel i.v./i.p, S1 oral	-	32.5	-	24% ^#^	62.5% (venous, arterial resection)	75%	16.3 months ^E^ 27.8 months ^S^
Frigerio [69]	2017	Bi-centric, retrospective	355 ^#^	NA	2007–2015	100%	0%	0%	FFX, Gem+ Nab-Placitaxel, Gem	-	32	95.5% ^&^	4.5% ^#^	8.3% (venous resection)	88%	56 months ^S^

NAT, neoadjuvant therapy; # intention-to-treat population (ITT); ^§^, weeks; HEP, hepatic metastasis; PUL, pulmonal metastasis; PER, peritoneal metastasis; S1, Tegafur with 5-chloro-2,4-dihydroxypyridine and potassium oxonate; FFX, FOLFIRINOX; Gem, Gemcitabine; *, including liver (*n* = 9) and lung resection (*n* = 2); ^$^, radiofrequency ablation (*n* = 1); E, Entire cohort; S, Surgical resected cohort.
